# The Effects of Toothbrush Wear on the Surface Roughness and Gloss of Resin Composites with Various Types of Matrices

**DOI:** 10.3390/dj9010008

**Published:** 2021-01-12

**Authors:** Murtadha AlAli, Nikolaos Silikas, Julian Satterthwaite

**Affiliations:** 1 Prosthodontic Department, Alahsa Dental Centre, Ministry of Health, Alahsa 39182, Saudi Arabia; 2 Division of Dentistry, School of Medical Sciences, The University of Manchester, Manchester M13 9PL, UK; nikolaos.silikas@manchester.ac.uk (N.S.); julian.satterthwaite@manchester.ac.uk (J.S.)

**Keywords:** dentistry, restorative composite materials, wear, gloss, surface roughness, dimethylacylates, Bis-GMA

## Abstract

Objective: To evaluate and compare the surface roughness and gloss of a DMA-free composite and Bis-GMA-free composite with a DMA-based composite before and after toothbrushing simulation. Materials and Methods: Fifteen dimensionally standardised composite specimens of three nano-hybrid resin composites (Tetric EvoCeram, Admira Fusion, and Venus Diamond) were used. Five specimens from each composite were polished and then subjected to a toothbrushing simulator. Surface roughness (Ra) and gloss were measured before toothbrushing and after 5000, 10,000, 15,000, and 20,000 toothbrushing cycles. The data was analysed using 5 × 3 ANOVA to assess surface roughness and gloss values and pairwise comparisons in the form of Tukey post hoc tests were performed to interpret main effects. Results: For all tested materials, surface roughness increased, and gloss decreased after toothbrushing abrasion. Surface roughness (Ra) values ranged from 0.14 to 0.22 μm at baseline and increased to between 0.41 and 0.49 μm after 20,000 toothbrushing cycles. Gloss values ranged between 31.9 and 50.6 GU at baseline and between 5.1 and 19.5 GU after 20,000 toothbrushing cycles. The lowest initial Ra value was detected in Venus Diamond and the highest initial gloss value was detected in Tetric EvoCeram. Conclusions: Simulated toothbrushing abrasion led to an increase in surface roughness and a decrease in gloss for all tested materials. Venus Diamond had the smoothest surface and Tetric EvoCeram had the glossiest surface after polishing and following 20,000 cycles of toothbrushing abrasion. Admira Fusion demonstrated the roughest surface and had the lowest gloss values before and after toothbrushing abrasion.

## 1. Introduction

Resin composites are the natural choice of restorative material for most dentists due to their aesthetic and mechanical properties [1]. Advances in formulations and technology have increased their use. Resin composite materials are available with different matrix formulations and different filler types that influence both their handling characteristics and physical properties [2,3]. One of the most important features to consider when selecting a resin composite is surface characteristics, such as surface roughness and gloss. The surface texture of dental materials has a significant effect on plaque accumulation, discoloration, wear, and appearance of direct and indirect restorations [4]. Moreover, a smooth surface increases patient comfort as any change in surface roughness between 0.30 μm and 0.50 μm can be detected by the tip of the tongue [5].

Resin-based composites are composed of an organic matrix consisting of one or more monomers; inorganic fillers and coupling agents; and other components such as initiators, accelerators, photo-initiators, photosensitisers, and polymerisation inhibitors [6]. Technical advances in filler technology (such as nano-hybrid composites) and matrix formulations (e.g., Ormocer and Bis-GMA-free monomers) have broadened the scope of resin composites available to clinicians [7]. However, there are few studies evaluating the surface properties of ormocer-based composites and Bis-GMA-free composites [8,9]. This study was therefore undertaken to compare the initially achieved surface roughness and gloss values following multiple cycles of toothbrushing abrasion of two new composite materials, Admira Fusion (a nano-hybrid Ormocer based composite) and Venus Diamond (a nano-hybrid TCD-Urethane based composite) compared to Tetric EvoCeram (a nano-hybrid DMA based composite). These materials were selected because they are based on different resin matrices with the same filler type and very similar filler loads which will allow a comparison of the effect of different resin matrix compositions on surface characteristics while taking into consideration the filler effect. 

The specific objective of this study was to evaluate and compare the changes in surface roughness and gloss of a DMA-free composite and a Bis-GMA-free composite with a dimethylacrylate-based composite before and after toothbrush simulation. The null hypothesis was that there is no significant difference in surface roughness and gloss before or after toothbrushing or between materials.

## 2. Materials and Methods

### 2.1. Materials Used in This Study

Three nano-hybrid-resin-based composite materials were used in this study (Table 1):

1. Tetric EvoCeram (TEC) (Ivoclar Vivadent, Schaan, Liechtenstein, shade A2, batch no W10431): based on dimethacrylate and Bis-GMA.
2. Admira Fusion (AD) (Voco, Cuxhaven, Germany, shade A2, batch no 1905236): based on ormocer as an example of a non-DMA group.
3. Venus Diamond (VD) (Kulzer, Hanau, Germany, shade A2, batch no K010072): as an example of a Bis-GMA-free group.

The Blue Sof-Lex disc polishing system (3M ESPE Dental Products, St. Paul, MN, USA) and Colgate toothpaste (Colgate-Palmolive Company, Guildford, UK) were also used. 

### 2.2. Methods

#### Specimen Preparations

Using a polytetrafluoroethylene (PTFE) mould, cylindrical specimens with dimensions of 15 mm × 2 mm were prepared. For each material, five specimens (*n* = 15) were prepared and the sample size calculation was done based on the results of the study by O’Neill et al., [9] (When performing sample size calculations, we made a conservative assumption of an effect size of f = 0.80, this reflecting the very large effects commonly found in studies comparing dental materials—for example, conservative calculations based on the descriptive statistics in the study by O’Neill et al. [9], yield effect sizes well in excess of 1.00 for measures of both gloss and roughness. Thus, assuming an effect size of f = 0.80, desired power of 80%, alpha of 0.05, and a reasonably large correlation (r = 0.70) among repeated measures, use of GPower for mixed 5 × 3 ANOVA suggested a total sample size of 15 (*n* = 5 for each of the three materials compared). A glass slide 1–2 mm thick was placed at the bottom of the mould filled by the composite material. A Mylar transparent strip was positioned on the top of each composite material and a glass slide was placed on it with pressure applied to it before curing. The specimens were then cured for 20 s at a distance of 1 mm according to manufacturers’ instructions using an Elipar S10 LED (3M ESPE, St. Paul, MN, USA) hand-held light cure and each specimen was cured from different positions to ensure homogenous polymerisation. The light cure had an output of 1200 mW/cm^2^ and a wavelength range of 430–480 mm as stated by the manufacturer. Following the preparation, the specimens were stored dry in a plastic bag at 37 °C for 24 h before the finishing and polishing procedures [10,11].

### 2.3. Finishing and Polishing

All specimens were ground wet with abrasive P500 grit silicon carbide (SiC) papers to remove any surface irregularities and to standardise the baseline. The specimens were then stored in plastic bags at room temperature for 24 h before testing. A metal digital calliper (Tachlife DC04 digital calliper, Shenzhen, China) was used to measure the width and thickness of each specimen.

All specimens were finished and polished using blue Sof-Lex^TM^ finishing and polishing discs (3M ESPE Dental Products, St. Paul, MN, USA) with coarse, medium, fine, and super-fine grits for 30 s each (timed using a stopwatch). The polishing procedure was performed using a low-speed handpiece at a speed of 12,000 rpm with a constantly moving repetitive stroking action. To achieve smooth and glossy surfaces and to create identical specimens with standardised baselines, each specimen was polished with a new set of Sof-Lex discs. After polishing, all specimens were cleaned for five minutes using an ultrasonic water bath (Ultrasonic cleaner L & R 2014, Kearny, NJ, USA).

### 2.4. Surface Roughness Measurement (Ra)

The surface roughness measurement was evaluated quantitatively using a profilometer (Talysurf CLI 1000, Taylor Hobson precision, Leicester, UK) which had a confocal point gauge based on the chromatic length aberration (CLA) principle (for non-contact high resolution measurement) [4]. The surface roughness of each specimen was evaluated before and after toothbrushing simulation by placing each specimen over a flat surface above cross-slides. It was then scanned by a confocal optical single point sensor with a 1 mm scanning length and a cut-off length of 0.25 mm. The mode of measurement was bidirectional gauge measurement, and the measurement speed was 500 μm/s. Data was obtained and analysed by TalyMap analysis software (Ultra version 6, Talymap Gold, Taylor Hobson, Leicester, UK) [12]. In this study, Ra was used as the mean measurement of the surface roughness as per ISO 4287. 

### 2.5. Surface Gloss Measurement 

The surface gloss of each specimen was recorded using a Novo-Curve gloss meter (Rhopoint Instrumentation LTD, Bexhill-on-Sea, UK). Gloss was measured by having a beam of light directed at an angle of 60° to the surface of the tested materials [13]; the light reflected at the same angle was then quantified. Prior to measuring the gloss, the device was calibrated against a black glass provided by the manufacturer with a reference value of 93.7 gloss units (GU). During the measurement of gloss, the specimens were positioned on the top plate of the gloss meter and covered with a black cover to avoid exposure to external light. Five measurements were recorded at the centre of each specimen, and the mean value was obtained for each specimen. Gloss measurements were recorded at baseline and after each cycle of toothbrushing simulation. 

### 2.6. Toothbrushing Simulation 

A custom-made toothbrush simulator based at the University of Manchester Dental Biomaterials Laboratory was used to simulate toothbrushing of specimens. Regular-headed toothbrushes (Oral-B 40 indicator, regular, Oral-B Laboratories, London, UK) were attached to the simulator holder and the toothbrush was positioned parallel to the specimen with the bristles in contact with the composite specimens. A commercial toothpaste (Colgate Total, Colgate-Palmolive, Guildford, UK) was mixed with water to prepare a slurry according to ISO/TS 1469-1 (2:1, water: toothpaste). The slurry was poured into the station of the toothbrushing machine and replaced for every four new specimens.

The applied load during simulated toothbrushing was 2.5 N as per the ISO standard specification (ISO 2813, 2014). A horizontal cross-toothbrushing technique was applied to each toothbrush head. A counter attached to the machine counted the number of movements at a speed of 78 cycles per minute. All specimens underwent 20,000 cycles. Surface roughness and gloss values were measured after 5000, 10,000, 15,000, and 20,000 cycles. Following each cycle of toothbrushing simulation and before surface roughness and gloss measurement were obtained, specimens were cleaned in an ultrasonic water-bath (Ultrasonic cleaner L & R 2014, Kearny, NJ, USA) to remove any potential slurry debris.

### 2.7. Statistical Analyses

Data was analysed using SPSS software (Version 23.0, IBM SPSS Inc., New York, NY, USA). Normality of the data was tested by the Shapiro–Wilk test, which revealed normal distribution (*p* > 0.05). A mixed 5 × 3 ANOVA with main effects and interactions was applied to assess surface roughness and gloss values at a pre-set *p* value of 0.05. A simple effects analysis was used to interpret interactions where they existed and pairwise comparisons in the form of Tukey post hoc tests were performed to interpret main effects. One-way ANOVA was also performed to identify any significant difference in surface roughness between the materials at baseline. Pearson’s correlation analysis was performed to investigate the linear relationships between surface roughness and gloss. For all analyses, a *p*-value of <0.05 was considered to be statistically significant.

## 3. Results

### 3.1. Surface Roughness Measurement (Ra)

**Figure 1:**
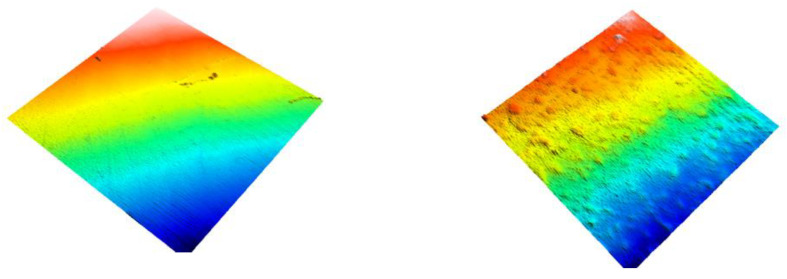
3D image of Tetric EvoCeram showing the surface of the resin-composite at baseline (**left image**) and after 20,000 cycles of toothbrushing abrasion (**right image**).

**Figure 2:**
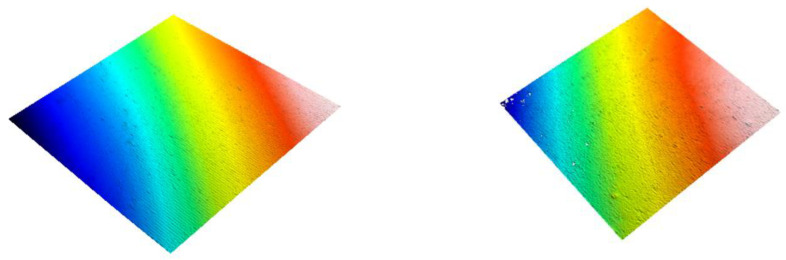
3D image of Venus Diamond showing the surface of the resin-composite at baseline (**left image**) and after 20,000 cycles of toothbrushing abrasion (**right image**)

**Figure 3:**
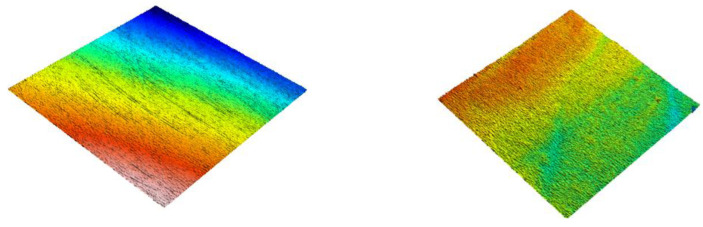
3D image of Admira Fusion showing the surface of the resin-composite at baseline (**left image**) and after 20,000 cycles of toothbrushing abrasion (**right image**). Note the surface irregularities at baseline

Figure 1, Figure 2 and Figure 3 show 3D scans of the composite specimens at baseline and after 20,000 cycles of toothbrushing abrasion.

The mean Ra values for the composite materials at baseline and after different numbers of toothbrushing cycles are described in Table 2. Surface roughness at the baseline was 0.14–0.22 μm, increasing to 0.41–0.49 μm following 20,000 cycles of toothbrushing abrasion. There was a significant main effect for the between-groups materials independent variable (*p* < 0.001) indicating a significant effect of material type on surface roughness. Moreover, the number of toothbrushing cycles had a significant effect on surface roughness (*p* < 0.001). However, the number of cycles by material type interaction was not significant (*p* > 0.05). Venus Diamond (VD) demonstrated the lowest Ra value at baseline, but there was no statistically significant difference with Tetric EvoCeram (TEC), whereas Admira Fusion (AD) showed the highest Ra value and was significantly different from both VED and TEC (*p* < 0.001) (Table 2). 

**Figure 4:**
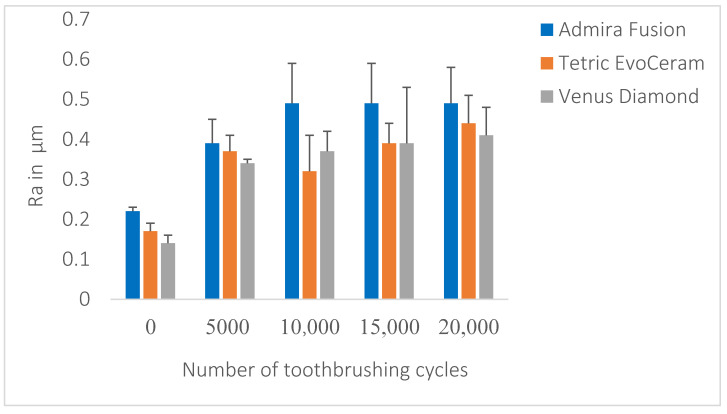
Ra values of the tested composite materials at baseline and after each set of 5000 cycles of toothbrushing abrasion.

Following toothbrushing abrasion, all tested materials demonstrated a significant increase in Ra values. However, after 20,000 cycles of toothbrushing abrasion, the overall Ra mean for AD was not significantly different from TEC, but it was significantly different from VED (*p* < 0.001). TEC was also not significantly different from VED (*p* < 0.001) (Table 2); this was confirmed by pairwise comparison and Tukey post hoc tests. The mean values for Ra after polishing and after 5000, 10,000, 15,000, and 20,000 toothbrushing cycles are illustrated in Figure 4.

### 3.2. Surface Gloss Measurement

Surface gloss at the baseline ranged between 31.9 and 50.6 gloss units (GU); this was reduced to 5.1–19.5 GU after 20,000 cycles of toothbrushing abrasion. All the materials demonstrated a significant decrease in the gloss values following 5000 cycles of toothbrushing abrasion (Table 3).

There was a significant between-materials effect (*p* < 0.001), indicating that the type of material had a significant effect on surface gloss. A significant within-cycles effect was also noted, indicating that the number of brushing cycles had a significant effect on surface gloss (*p* < 0.001). Moreover, a significant cycles-by-material type interaction was identified (*p* < 0.001). 

TEC showed the highest gloss value at the baseline with a significant difference (*p* < 0.001) from both VED and AD. AD had the lowest gloss value from the baseline to 20,000 cycles of toothbrushing abrasion; this was statistically different from that of TEC and VED, but the reduction in gloss value from 5000 cycles to 20,000 cycles was not statistically significant for AD. 

**Figure 5:**
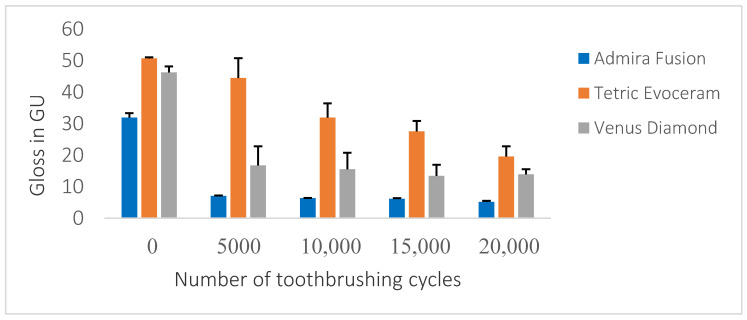
Gloss values for each of the tested composites at baseline and after each set of 5000 cycles of toothbrushing abrasion.

TEC showed a significant decrease in gloss values among all the cycles of toothbrushing abrasion, while VED demonstrated no significant difference in gloss values (*p* > 0.05) between 5000, 10,000, and 20,000 cycles of toothbrushing abrasion. However, at 15,000 cycles, the change in gloss value was statistically different from 5,000 and 10,000 cycles (*p* < 0.001), but then there was no significant difference at 20,000 cycles (*p* > 0.05) (Figure 5).

### 3.3. The Association between Surface Gloss and Roughness 

Pearson’s correlation test showed a negative linear relationship between surface roughness and gloss for all tested materials, meaning that the lower surface roughness is the higher the gloss is and vice versa. For example, at 15,000 cycles, TEC, and VED showed high gloss values and low surface roughness values. On the other hand, AD showed a low gloss value and a high surface roughness value. The strength of this association ranged from weak to moderate (0.1 < |*r*| < 0.7) *p* < 0.001.

## 4. Discussion

The surface roughness and gloss of any resin composite material are the products of the interaction of several intrinsic and extrinsic factors. Intrinsic factors are related to the material itself e.g., type of resin matrix, the filler (type, size, and distribution of the particles) and the effectiveness of the bond at the filler/resin interface [14,15]. Extrinsic factors are related to the type of polishing system used and the light-curing method. In this study, the polishing system and light-curing method were standardised for all the tested materials, and all tested materials were nano-hybrids with their filler loadings being similar to each other. 

Following the polishing and finishing procedures, the average surface roughness (Ra) values ranged from 0.14 to 0.22 μm and gloss values ranged from 31.9 to 50.6 GU. The Ra values demonstrated by VED and TEC were very similar with no statistically significant difference, whereas AD had a significantly rougher surface than the other two materials. The highest gloss value was demonstrated by TEC and the lowest value by AD. The particle size of a composite material influences its polishability: the smaller the particle size the smoother and glossier will the surface be after polishing [16,17]. 

All tested materials contained nano-hybrid particles that combined nanometric and conventional fillers with a comparable average particle size. It was thus expected that they would show comparable Ra and gloss values following the polishing procedures. However, variations in Ra and gloss values were found, which could be related to the fact that TEC and VED had a relatively lower filler content by weight (78–80%) compared to AD which had an 84% filler content by weight. This could have affected both Ra and gloss values. Furthermore, the distribution of filler particles has an effect on surface roughness and gloss, whereby a material that contains more nano-sized fillers produces a lower Ra value [16]. The difference may therefore be attributable to the distribution of the particles with the possibility that the whole size distribution of VED is skewed to the small particle size, thus showing the smoothest surface. In addition, VED has a smaller nanometric particle size of 5 nm compared to 40 nm for AD and TEC.

Resin matrix composition is also a factor that has a significant effect on Ra and gloss. VED is based on TCD-urethane, which shares the same properties as Bis-GMA, the resin matrix for TEC. The backbone of both resins has been proven to be rigid [18], and this could explain why both VED and TEC showed similar Ra values after polishing. The resin may not have been completely removed after polishing, and thus less filler was exposed, leading to lower Ra and higher gloss values. Furthermore, the higher degree of monomer conversion in VED [19] might be an additional factor for the low roughness value; this has been proven to have an effect on surface roughness [18,20]. TEC also contains “pre-polymers”, which are a pre-cured composite, i.e., organic matrix and inorganic fillers that are milled to produce filler particles with a desired grain size [21]. The finished composite material consists of the pre-polymers mixed with additional filler and monomer. The pre-curing of these prepolymers may increase the degree of conversion of monomer, which might be reflected in lower roughness values. Ormocers (standing for organically modified ceramics) are produced by combining organic-inorganic co-polymers with ceramic materials. The combination of the complex network matrix and larger filler particles in AD may have resulted in an inhomogeneous structure following the polishing procedures [22,23], leading to different wear values between the resin and the filler. This could have been responsible for the increase in surface roughness and the decrease in gloss values [23]. This may therefore indicate that ormocer is a soft resin that can be worn away easily following polishing; this would lead to a rougher and a less glossy surface than the other tested materials. 

Simulated toothbrushing abrasion significantly affected both surface roughness and gloss. For all tested materials, Ra significantly increased and gloss significantly decreased. All the materials showed similar increases in Ra values with no significant difference between AD and TEC and between VED and TEC following the 20,000 cycles of toothbrushing abrasion, corresponding to 4–7 years of toothbrushing [24]. This is in line with another study that compared Ra before and after toothbrushing abrasion of ormocer-based composites with composites based on conventional monomer systems [8].

There was a significant difference in gloss values after toothbrushing abrasion between all the materials, with TEC having the highest gloss values and AD the lowest. The action of toothbrushing creates both macroscopic and microscopic irregularities on the surface of teeth, and this increase in Ra reflects light in irregular patterns and reduces gloss [25]. In addition to surface roughness, gloss can be influenced by changes in the refractive indices of the matrix and the filler [26]. For AD, the increase in Ra values and decrease in gloss values could be related to the deficiency in the cohesion between the novel resin matrix (ormocer) and the fillers during the silanisation process that may lead to particles of filler being dislodged from the resin matrix during toothbrushing abrasion. Additionally, when larger filler particles are abraded there is a consequential loss of resin and this causes an increase in the Ra value and a decrease in gloss [27]. 

TEC has a pre-polymerised filler content of 34%. These have fewer residual double bonds that lead to weaker bonds with the resin matrix and consequently to failure of that interface. In addition, pre-polymerised fillers do not silanise easily and cannot create good binding sites, and thus do not integrate easily into the resin matrix; this leads to an increase in Ra values after toothbrush abrasion [9]. For VED, the increase in Ra values may be due to the weak silane coupling between the resin matrix and the fillers along with the wide filler size range (up to 20 μm). As a result of the weak bond, some of the large fillers might have detached from the matrix, resulting in concavity in the surface following toothbrushing abrasion, thereby increasing the surface roughness and decreasing the surface gloss [28]. Many studies concluded that toothbrushing significantly increases surface roughness [24,27] and any surface roughness above a threshold of 0.2 μm increases bacteria retention [29]. Therefore, the surface of a restoration is of considerable importance because it affects the function of a tooth through wear, abrasiveness, and build-up of plaque and calculus [30]. A smoother restoration has a better wear resistance and marginal integrity thus increasing the life of the restoration and the oral health of the patient [4]. Wear is a complex mechanism involving abrasion, adhesion, erosion, fatigue, and friction [31] and wear due to toothbrushing abrasion has been shown to affect both optical and structural properties of dental materials resulting in a remarkable impact on aesthetic [32] and hardness of the resin composites [33]. The enamel wear can also be increased by a considerably rough surface [34] and the findings of this study confirmed that simulated toothbrushing abrasion significantly increased surface roughness and decreased surface gloss and this has a clinical significance as a rougher and less glossy composite might result in undesirable biological and aesthetic effects [15]. 

With regards to gloss, TEC had the glossiest surface after abrasion with a toothbrush even though it did not have the smoothest surface. This could be related to the distribution range of filler particles, with smaller particles being more dominant in TEC after the abrasion. Another factor that could be related to the resin matrix is that TEC is based on Bis-GMA, which has a large molecule size and chemical structure, and this produces stronger and stiffer resins [35]. Bis-GMA resin may thus not be removed easily following abrasion and this would leave fewer filler particles to exfoliate. However, the mean gloss values for AD were not significantly different after 5000 cycles as the gloss value was already low at 5000 cycles (7 GU). The possible explanation for this is that the first toothbrushing cycle removed the highly polished surface created by Sof-lex discs and the layer exposed following the toothbrushing cycles was more resistant to increasing toothbrushing cycles. This is clinically relevant, as the surface gloss of AD was not significantly reduced after the removal of the polished surface by toothbrushing. 

VED showed no significant difference in gloss values after increasing the toothbrushing cycles to 5000, 10,000, and 20,000, but at 15,000 there was a significant difference in gloss values in comparison to 5000 and 10,000, but not with 20,000. This is clinically relevant, as VED showed similar behaviour with no significant decrease in gloss values following the removal of the polished layer by toothbrushing. However, this was not constant, and progressive reduction in gloss values was seen following increased toothbrushing abrasion. These findings suggest that TEC and VED become less glossy over time, and their gloss was negatively affected by increasing the number of toothbrushing cycles.

Negative correlations between Ra and gloss values have been reported in many studies [36,37] and this was also confirmed by this study. Surface roughness and gloss are influenced by similar factors such as the resin matrix, filler type, and the quality of the bond between them [15]. This could explain the negative correlation between them. This study found that TEC had the glossiest surface after abrasion with a toothbrush even though it did not have the smoothest surface. It can be concluded that the composition of the material may have a greater effect on glossiness than does roughness [37]. Concerning the limitations of the present study, it is worth noting that although the methods used in this study were standard and all the tested materials were nano-hybrid composites with similar filler loadings, there was a variation in filler shape, weight, and size because commercial resin composites were used; these filler properties affect surface characteristics. The resin matrix could thus not have been the only variant between the materials. In addition, surface roughness and gloss have been evaluated only quantitatively in this study and this hindered the assessment of surface changes qualitatively. In addition, the deterioration process and the changes in surface characteristics (Ra and gloss) in this study did not consider patient-related factors such as saliva and mucosa, which may influence the absolute values after toothbrushing. 

## 5. Conclusions

Tested composite materials based on methacrylate resin and its derivatives show better surface roughness and gloss characteristics compared to restorative composite materials based on non-DMA resin matrices that may have a greater biocompatibility.

## Figures and Tables

**Table 1:** List of the materials used in this study.

Material	Manufacturer	Type	Code	Shade	Monomer	Composition Filler	wt.%/vol%	Batch No.
Tetric EvoCeram	Ivoclar Vivadent (Schaan, Liechtenstein)	Nano-hybrid composite	TEC	A2	Dimethacrylates Bis-GMA	Barium glass, ytterbium trifluoride, mixed oxide and copolymers (40 nm and 3 μm) (0.6 μm average)	78/55	W10431
Admira Fusion	Voco (Cuxhaven, Germany)	Nano-hybrid ormocer-based Composite	AD	A2	3-dimensionally linked inorganic organic copolymers (Ormocers)	Silicon oxide, glass-ceramic filler (<1 μm) (0.04–1.2 μm, average 0.7 μm)	84/60	1905236
Venus Diamond	Kulzer (Hanau, Germany)	Nano-hybrid Composite	VED	A2	TCD-urethane cross linker (TCD-DI-HEA and UDMA)	Barium aluminium fluoride Glass Highly discrete nanoparticles (5 nm–20 μm)	80/65	K010072
Colgate Cavity Protection	Colgate-Palmolive (Guildford, UK)	Toothpaste			Sodium fluoride (450 ppm F), sodium monofluorophosphate (1000 ppm F), natrium fluoride (450 ppm F), natrium monofluorphosphate (100 ppm F)			T8014003

**Table 2:** Means and Standard deviations of Ra values in μm for the composite materials after different numbers of toothbrushing cycles.

Material	0 Cycle	5000 Cycles	10,000 Cycles	15,000 Cycles	20,000 Cycles	Mean
Admira Fusion	0.22 ± 0.01	0.39 ± 0.06	0.49 ± 0.1	0.49 ± 0.10	0.49 ± 0.09	0.42 ± 0.14 ^a^
Tetric EvoCeram	0.17 ± 0.02	0.37 ± 0.04	0.32 ± 0.09	0.39 ± 0.05	0.44 ± 0.07	0.34± 0.10 ^a,b^
Venus Diamond	0.14 ± 0.02	0.34 ±0.01	0.37 ± 0.05	0.39 ± 0.14	0.41 ± 0.07	0.33 ± 0.12 ^b^
Mean	0.18 ± 0.04	0.37 ± 0.05 ^a^	0.40 ± 0.11 ^a,b^	0.42 ± 0.11 ^a,c^	0.45 ± 0.08 ^b,c^	

*n* = 5 specimens per group. Mean surface roughness values that are not significantly different are indicated by the same lowercase superscript letter (post hoc analysis Tukey HSD *p* > 0.05).

**Table 3:** Mean gloss values and standard deviations for the tested materials after different numbers of toothbrushing cycles.

Material	0 Cycles	5000 Cycles	10,000 Cycles	15,000 Cycles	20,000 Cycles
Admira Fusion	31.9 ± 1.4	7.0 ± 0.2 ^a^	6.3 ± 0.1 ^a^	6.1 ± 0.2 ^a^	5.1 ± 0.4 ^a^
Tetric EvoCeram	50.6 ± 0.36	44.4 ± 6.3	31.9 ± 4.5	27.5 ± 3.3	19.5 ± 3.3
Venus Diamond	46.2 ± 1.9	16.7 ± 6.1 ^b^	15.5 ± 5.2 ^b^	13.4 ± 3.5 ^c^	13.9 ± 1.6 ^b,c^

*n* = 5 specimens per group. Mean surface gloss values that are not significantly different are indicated by the same lowercase superscript letter (Simple Effect Analysis *p* > 0.05).

## Data Availability

Not applicable.

## Clinical Significance

This study has proved that tested composite materials based on methacrylate resin matrix and its derivatives possess better surface roughness and gloss properties compared to restorative composite materials based on non-DMA resin matrices that may have a greater biocompatibility.
